# Prophylactic Vancomycin Drops Reduce the Severity of Early Bacterial Keratitis in Keratoprosthesis

**DOI:** 10.1371/journal.pone.0139653

**Published:** 2015-10-13

**Authors:** Aris Konstantopoulos, Xiao Wei Tan, Gwendoline Tze Wei Goh, Padmanabhan Saraswathi, Liyan Chen, Chan Lwin Nyein, Lei Zhou, Roger Beuerman, Donald Tiang Hwee Tan, Jod Mehta

**Affiliations:** 1 Singapore Eye Research Institute, Singapore; 2 Singapore National Eye Centre, Singapore; 3 Department of Clinical Sciences, Duke-NUS Graduate Medical School, Singapore; 4 Nanyang Technological University, Singapore; University of Missouri-Columbia, UNITED STATES

## Abstract

**Background:**

Artificial cornea transplantation, keratoprosthesis, improves vision for patients at high risk of failure with human cadaveric cornea. However, post-operative infection can cause visual loss and implant extrusion in 3.2–17% of eyes. Long-term vancomycin drops are recommended following keratoprosthesis to prevent bacterial keratitis. Evidence, though, in support of this practice is poor. We investigated whether prophylactic vancomycin drops prevented bacterial keratitis in an animal keratoprosthesis model.

**Methodology:**

Twenty-three rabbits were assigned either to a prophylactic group (n = 13) that received vancomycin 1.4% drops 5 times/day from keratoprosthesis implantation to sacrifice, or a non-prophylactic group (n = 10) that received no drops. All rabbits had *Staphylococcus aureus* inoculation into the cornea at 7–12 days post-implantation and were sacrificed at predetermined time-points. Prophylactic and non-prophylactic groups were compared with slit-lamp photography (SLP), anterior segment optical coherence tomography (AS-OCT), and histology, immunohistochemistry and bacterial quantification of excised corneas. Corneal vancomycin pharmacokinetics were studied in 8 additional rabbits.

**Results:**

On day 1 post-inoculation, the median SLP score and mean±SEM AS-OCT corneal thickness (CT) were greater in the non-prophylactic than the prophylactic group (11 vs. 1, p = 0.049 and 486.9±61.2 vs. 327.4±37.1 μm, p = 0.029 respectively). On days 2 and 4, SLP scores and CT were not significantly different. Immunohistochemistry showed a greater CD11b+ve/non-CD11b+ve cell ratio in the non-prophylactic group (1.45 vs. 0.71) on day 2. Bacterial counts were not significantly different between the two groups. Corneal vancomycin concentration (2.835±0.383 μg/ml) exceeded minimum inhibitory concentration (MIC) for *Staphylococcus aureus* only after 16 days of vancomycin drops. Two of 3 rabbits still developed infection despite bacterial inoculation after 16 days of prophylactic drops.

**Conclusions:**

Prophylactic vancomycin drops provided short-term benefit, but did not prevent infection. Achieving MIC in the cornea was not sufficient to prevent *Staphylococcus aureus* keratitis. Patients should continue to be counselled regarding the risk of infection following keratoprosthesis.

## Introduction

Corneal infection and inflammatory disorders are significant causes of global visual impairment and blindness. According to the World Health Organisation, corneal disease predominantly in association with infection and scarring is a major cause of blindness in the world, second only to cataract.[1] Globally, 2.85 million people are estimated to be visually impaired and 1.56 million blind due to corneal opacities.[2] In addition to limited worldwide availability of high quality corneal material, the prognosis of corneal grafts, even in developed countries, is relatively poor in the presence of ocular surface disease, inflammation or failed previous graft.[3,4] Artificial corneas have the potential to eliminate immune mediated rejection and failure, especially in higher risk cases.

Results with fully synthetic corneas have been poor;[5,6] the AlphaCor artificial cornea had a retention rate of 80% and 62% at 1 and 2 years respectively.[5] Devices that use a hybrid of synthetic and biological tissue, such as the Boston keratoprosthesis (Kpro), have recently gained in popularity, as they provide visual improvement with a low extrusion rate.[7–9] In a large multi-centre study, the Boston type 1 retention rate was 67% at 7 years.[10] Limitations, however, such as the requirement for cadaveric cornea, the development of glaucoma, retroprosthetic membrane and infection limit more widespread use.[7,11–13]

Infection in Kpro is a devastating complication that can cause acute deterioration of vision. Published rates vary depending on follow-up, ranging between 3.2–17% of eyes.[14–17] Prophylactic vancomycin drops following Boston Kpro have been accredited with a reduction in rate of bacterial endophthalmitis.[18,19] However, they have not been found to reduce bacterial keratitis rates in Boston Kpro.[16] Studies on Kpro associated infection should be interpreted within the context of their retrospective nature, small cohorts and heterogeneity of patients and causative microbial pathogens.

In this prospective study we investigated whether regular application of vancomycin drops could prevent the development of *Staphylococcus aureus* keratitis in an established animal model of Kpro associated microbial keratitis.[20]

## Materials and Methods

The study adhered to the Statement for Use of Animals in Ophthalmic Vision and Research by the Association for Research in Vision and Ophthalmology. The protocol was approved by the Institutional Animal Care and Use Committee and Institutional Biosafety Committee at Singapore Eye Research Institute. Thirty-one New Zealand White rabbits were used.

### Rabbit model

New Zealand White rabbits, aged 1 to 2 months and weighing 2 to 2.5 kg, were anaesthetised using an intramuscular injection of ketamine hydrochloride (35 mg/kg; Parnell Laboratories, Alexandria, Australia) and xylazine hydrochloride (5 mg/kg; Troy Laboratories, Smithfield, Australia). The right eye was chosen for surgery and a titanium film implanted, as previously described.[20] Briefly, a 7 mm diameter and 75% deep corneal stromal pocket was created using the Visumax^TM^ femtosecond laser (Carl Zeiss Meditec, Jena, Germany). A 5 mm wide superior arcuate incision was made by a guarded diamond blade (Storz, Bausch and Lomb, USA), the pocket opened up with a Seibel spatula (Rhein Medical Inc., Petersburg, FL) and a 4 mm diameter titanium film implanted into the pocket.

**Fig 1:**
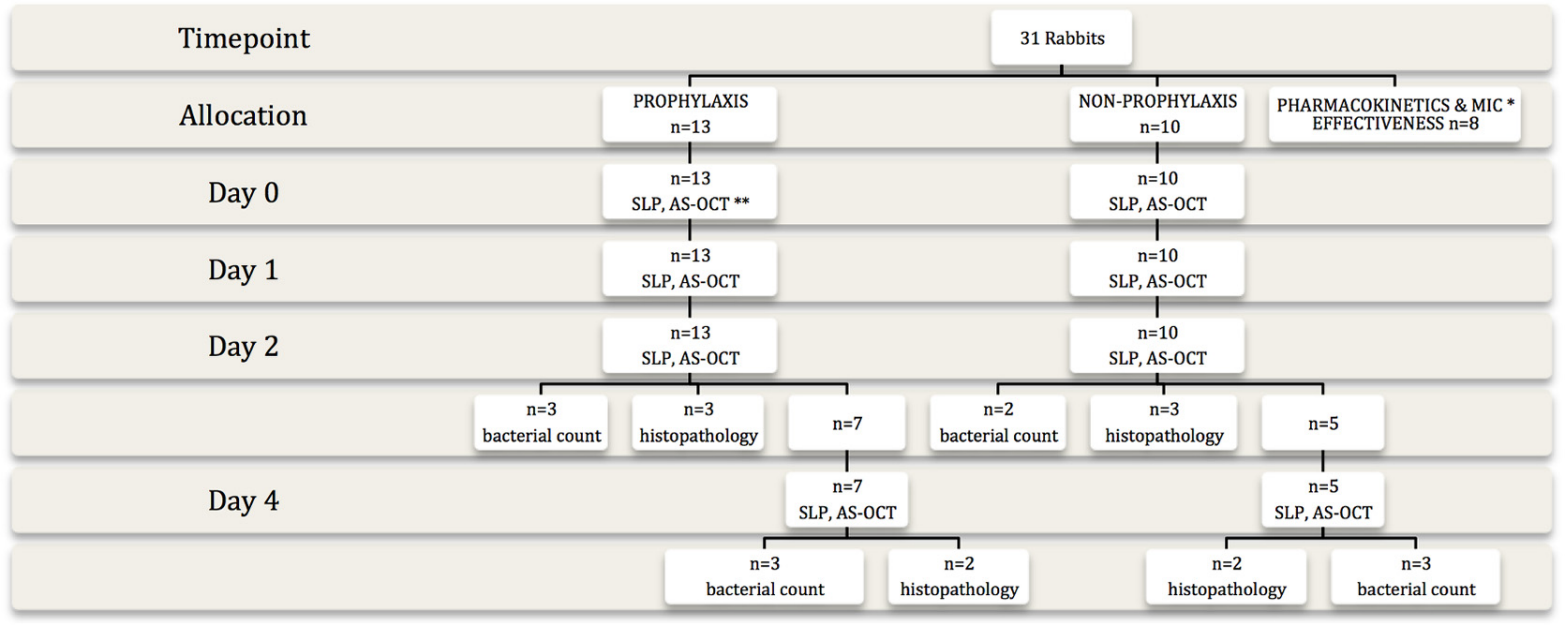
Study design. The flow chart demonstrates allocation to prophylactic and non-prophylactic groups, follow-up and investigations carried out at each time point. (* MIC: minimum inhibitory concentration, ** SLP: slit-lamp photography, AS-OCT: anterior segment optical coherence tomography)

Twenty-three rabbits, used for the prophylactic versus non-prophylactic study, had a 25 μl bacterial solution (*Staphylococcus aureus* ATCC29213, 2X10^4^ CFU/ml) inoculated with a 29 G needle into the corneal pocket above the titanium film at 7–12 days post-implantation and were sacrificed at predetermined time-points (Fig 1). Euthanasia was carried out with intravenous pentobarbitone (85 mg/kg) in anaesthetized rabbits.

Eight rabbits were used exclusively to study the corneal pharmacokinetics of vancomycin and the effectiveness of the corneal vancomycin minimum inhibitory concentration (MIC) for *S*. *aureus*; 3 rabbits without an implant were sacrificed after 4 days of bilateral vancomycin drop instillation, 2 rabbits with a titanium film implant were sacrificed after 10 days of vancomycin drop instillation and 3 rabbits with a titanium film implant had a 25 μl bacterial solution (*Staphylococcus aureus* ATCC29213, 2X10^4^ CFU/ml) inoculated 16 days post-implantation and were sacrificed once corneal infection had developed.

### Prophylactic versus non-prophylactic study

Twenty-three rabbits were assigned either to a prophylactic group or a non-prophylactic group. The prophylactic group (13 rabbits) received one drop of vancomycin 1.4% (Singapore General Hospital, SingHealth) to the right eye 5 times a day from titanium film implantation to sacrifice; 6 rabbits were sacrificed on day 2 post-inoculation and 7 on day 4 post-inoculation. The non-prophylactic group (10 rabbits) received no drops to the right eye after surgery; 5 rabbits were sacrificed on day 2 post-inoculation and 5 on day 4 post-inoculation.

Slit-lamp photography and anterior segment optical coherence tomography (AS-OCT) were carried out before and after bacterial inoculation. The corneas of sacrificed rabbits were examined for histology, immunohistochemistry and quantification of viable bacteria. The clinical imaging and laboratory parameters were compared between prophylactic and non-prophylactic groups. The study design is illustrated in Fig 1.

#### Slit lamp photography and anterior segment optical coherence tomography

**Fig 2:**
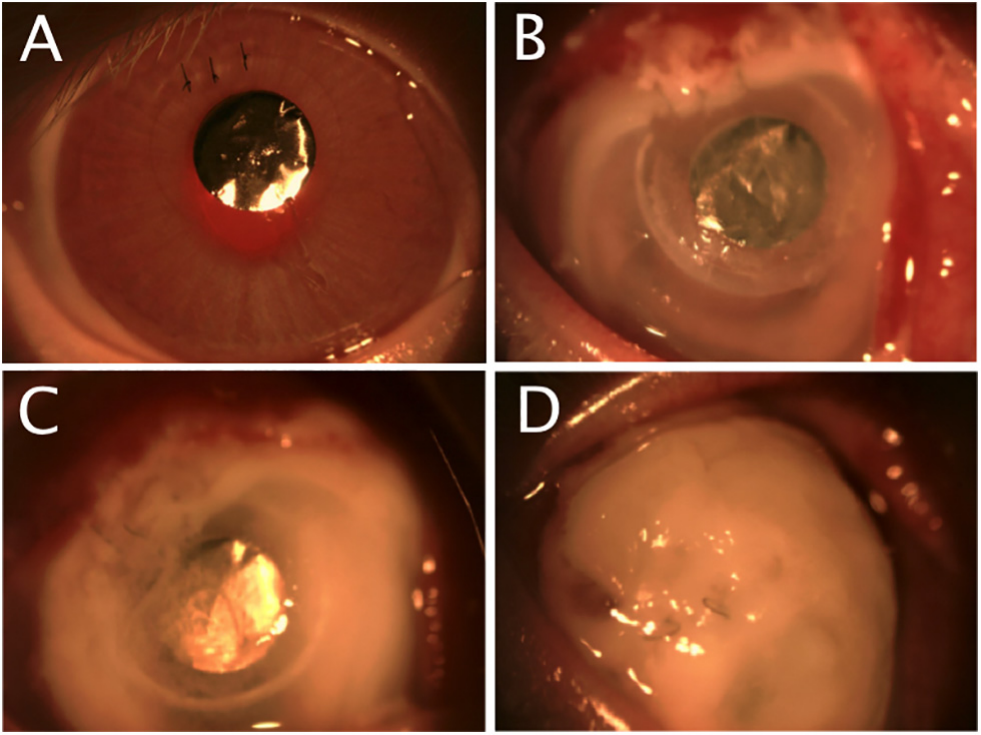
Serial slit-lamp photography for clinical grading. A. A quiet eye with a clear cornea and the titanium keratoprosthesis in-situ before bacterial inoculation. B. On day 1 following inoculation, the conjunctiva is injected and the cornea oedematous with early infiltration. C. On day 2, moderate corneal infiltration has developed. D. On day 4, severe corneal infiltration is present.

Slit-lamp photographs (Zoom Slit Lamp NS-2D, Righton, Tokyo, Japan) and AS-OCT scans (RTVue; Optovue, Inc, Fremont, CA) were taken before and after titanium film implantation, immediately before bacterial inoculation, and on days 1, 2 and 4 after bacterial inoculation. Serial slit-lamp photographs in Fig 2 illustrate the progression of infection.

Post-inoculation slit-lamp photographs were graded, using a condensed version of a previously described scale.[21] Briefly, the photos were graded in a blind manner using a scale of 0 to 4 for each of 4 parameters: conjunctival injection, conjunctival chemosis, corneal oedema and corneal infiltration. The individual parameter scores were added to give a total slit-lamp photography (SLP) score ranging from 0 to 16.

**Fig 3:**
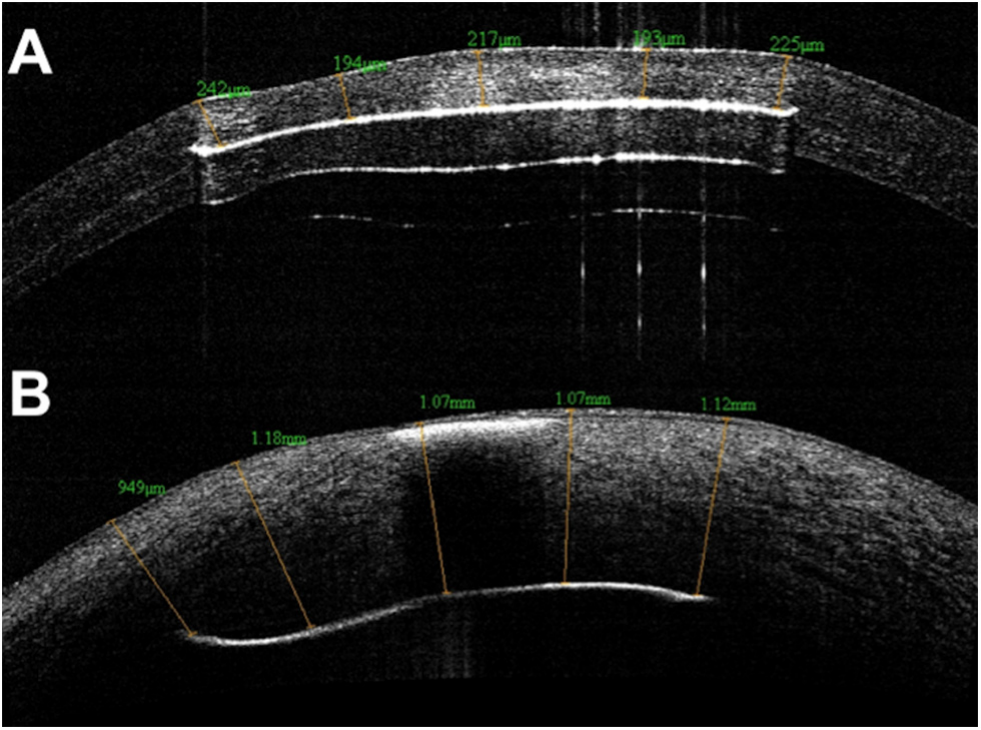
Anterior Segment Optical Coherence Tomography. Corneal thickness between implant and anterior corneal surface was measured at 5 locations before (A) and after (B) bacterial inoculation.

Pre-inoculation and post-inoculation corneal thickness (CT) measurements were carried out between the anterior corneal and implant surfaces on AS-OCT scans through the implant centre. Five measurements, all perpendicular to the anterior corneal surface, were carried out and the mean CT was calculated (Fig 3).

#### Quantification of viable bacteria

On days 2 and 4 post-inoculation, rabbit corneas were removed by trephination. They were individually homogenized in sterile phosphate buffered saline (PBS) using plastic pestles followed by fine homogenization with bead beating using sterile glass beads (2 mm). The homogenate then underwent serial dilution plating using Tryptic Soy Agar (TSA) plates (Beckman, USA). The plates were incubated at 35°C for 48 hours. The numbers of colonies were counted and the results expressed as log_10_ number of CFU/ cornea.

#### Histology and immunohistochemistry

Excised corneas of rabbits that were sacrificed on days 2 and 4 were fixed in 4% paraformaldehyde followed by dehydration with a serial concentration of ethanol. After dehydration, tissue blocks were embedded into paraffin and cut at 5 μm thickness using a microtome. The sections were stained with Hematoxylin (Sigma Aldrich, St. Louis, MO, USA) and Eosin (Sigma Aldrich, St. Louis, MO, USA) and then viewed under a light microscope.

Tissue blocks were also embedded in optimum cutting temperature (OCT) cryo-compound (Leica Microsystems, Nussloch, Germany) for immunohistochemistry studies. Frozen tissue blocks were stored at −80°C until sectioning. Serial sagittal corneal 10 μm sections were cut using a cryostat (Microm HM550; Microm, Walldorf, Germany). Sections were placed on polylysine-coated glass slides and air dried for 15 minutes.

For Hematoxylin and Eosin (H&E) staining, tissue sections were immersed in hematoxylin and eosin solutions for 10–20 seconds before cleaning with pure xylene. For immunohistochemistry, tissue sections were post-fixed with 4% paraformaldehyde for 15 minutes, washed with PBS and blocked with 10% normal goat serum in 1X PBS and 0.15% Triton X-100 for 1 hour. The sections were incubated with rat monoclonal antibody against CD11b (Abcam, SanFrancisco CA) diluted 1:100 at 4°C overnight. After washing with 1X PBS, the sections were incubated with goat anti-rat Alexa Fluor 488 conjugated secondary antibody (Invitrogen, Carlsbad, CA) at room temperature for 1 hour. Slides were then mounted with UltraCruz Mounting Medium containing 4′, 6-diamidino-2-phenylindole (DAPI; Santa Cruz Biotechnology, Santa Cruz, CA). For negative controls, non-immune serum was used in place of the specific primary antibody. Sections were observed and imaged with a fluorescence microscope (Carl Zeiss).

Microscopic qualitative assessment was carried out on H&E stained sections. Immunostaining for CD11b positive (+ve) cells was quantitatively compared between prophylactic and non-prophylactic cases. Five random corneal sections from each rabbit were examined for the ratio of CD11b positive/non-CD11b positive cells.

### Pharmacokinetics and effectiveness of minimum inhibitory concentration of vancomycin

Two rabbits with a titanium film implant had corneal and aqueous vancomycin quantification of the right eye after 10 days of instillation of one drop of vancomycin 1.4% 5 times a day. The left eye of 6 rabbits was used for corneal and aqueous vancomycin quantification; 3 rabbits had one drop of vancomycin 1.4% instilled 5 times a day for 10 days before sacrifice and 3 rabbits for 16 days.

Three rabbits had bilateral vancomycin quantification following 4 days of vancomycin 1.4% drop instillation 5 times a day. Prior to starting the antibiotic regimen, the cornea of the right eye had complete epithelial debridement following application of 20% alcohol for 60 seconds, whereas the epithelium of the left cornea was not debrided.

A further 3 rabbits with a titanium film implant to the right eye had bacterial inoculation, as described above, in order to investigate the effectiveness of vancomycin prophylaxis once the corneal vancomycin MIC for *Staphylococcus aureus* (2.0 μg/ml) had been achieved. Inoculation was carried out after 16 days of vancomycin 1.4% drop instillation 5 times a day, as corneal vancomycin quantification at the different time intervals showed that the MIC was achieved by day 16 of antibiotic use. Following bacterial inoculation, development of infection was assessed clinically with SLP and AS-OCT imaging.

#### Liquid Chromatography–Mass Spectrometry

Quantification of corneal vancomycin was carried out by Liquid Chromatography–Mass Spectrometry (LC-MS). Vancomycin standards (Vancomycin hydrochloride, Hospira, Lake Forest, Illinois, USA) were prepared by dissolution of a single dose vial and serial dilution with water. Aqueous humour samples (120–150 μL) were extracted with three equivalents of methanol (Merck, Darmstadt, Germany). The samples were shaken for 5 min at 1200 rpm (20°C) and centrifuged for 10 min at 16,000 g (4°C). The supernatants were transferred to fresh tubes and dried in a vacuum concentrator. Cornea samples were cut and ground with a pestle while frozen. They were next homogenized on ice with 300 μL of 3:1 methanol/water with 0.1% formic acid (Sigma Aldrich, St. Louis, MO, USA) for 1 min. The homogenates were then spun down briefly on a capsule centrifuge and supernatants transferred to fresh tubes. The residues were homogenized and spun down a second time. The extracts were combined and centrifuged for 10 min at 16,000 g (4°C). The supernatants were transferred to fresh tubes and dried in a vacuum concentrator. Samples were reconstituted in water (Ultrapure water, Millipore purification unit) and centrifuged for 5 min at 16,000 g (10°C) before transferring to autosampler vials for LC-MS/MS analysis.

Chromatographic separation was performed on a Waters 2695 Separations Module (Milford, MA, USA) with a Thermo Scientific Hypersil Gold C18 column (Whaltham, MA, USA) (2.1 × 50 mm, 3 μm). The mobile phase was A: 0.1% formic acid in water and B: 0.1% formic acid in acetonitrile. The gradient profile was 2% B at 0 min, 25% B at 6 min, 90% B from 6.5 to 8.5 min and 2% B from 9 to 13 min. The flow rate was 0.3 ml/min. The autosampler and column heater temperatures were maintained at 10 and 30°C, respectively. Detection was performed by an AB Sciex API 2000 triple quadrupole mass spectrometer (Concord, Canada) with an electrospray ionization source operating in the positive ionization mode. The ion source voltage was set to 5 kV. Vancomycin was detected by monitoring the transition 725.5/144.0 with collision energy of 25 V.

Calculation of the corneal vancomycin concentration was based on the amount of vancomycin detected in the cornea by LC-MS, the gross weight of the cornea and the assumptions that 1 ml of water weighs 1 gram and that the water content of the cornea was 78%.[22,23]

### Statistical analysis

Normality distribution of data was assessed by Shapiro-Wilk statistics and histograms. Mean or median values were compared between the prophylactic and non-prophylactic groups. The CT values were compared with the t-test and ANOVA, the total SLP scores with the Kruskal-Wallis and Mann-Whitney U-tests. Bacterial counts and vancomycin concentrations were compared with the t-test. The Statistical Package for Social Science (SPSS), version 15, was used; statistical significance was considered p<0.05.

## Results

### Slit-lamp photography

Overall, the median total SLP score increased from 4.0 (Inter-quartile range [IQR] 1,12) on day 1 to 12 (IQR 5,14.5) on day 2 and 16 (10,16) on day 4 (p = 0.004).

On day 1, the median total SLP score was greater in the non-prophylactic group compared to the prophylactic group (p = 0.049). On days 2 and 4, the scores were not significantly different between non-prophylactic and prophylactic groups (p = 0.456 and p = 0.527 respectively). The SLP scores are detailed in Table 1.

**Table 1:** Slit-lamp photography score comparison between prophylactic and non-prophylactic groups.

Parameter	Day	Prophylactic	Non-prophylactic	P value
		Slit-Lamp Photography score [median (IQR)]		
Conjunctival injection	1	0 (0, 3)	3 (0, 3.25)	0.166
Conjunctival chemosis	1	0 (0, 3)	3 (0.75, 3.25)	0.148
Corneal infiltration	1	0 (0, 1)	1.5 (0, 3)	0.232
Corneal oedema	1	1 (0, 3)	3 (1, 3.25)	0.115
**Total**	1	1 (0, 10.5)	11 (1, 13)	0.049
Conjunctival injection	2	2.5 (1, 3)	3 (1, 4)	0.539
Conjunctival chemosis	2	2.5 (1, 3)	3 (1, 4)	0.497
Corneal infiltration	2	2.5 (1, 3.75)	3 (1, 4)	0.418
Corneal oedema	2	3 (1.25, 4)	3.5 (2, 4)	0.628
**Total**	2	10.5 (4.25, 13.75)	12.5 (5.75, 16)	0.456
Conjunctival injection	4	4 (2, 4)	4 (2.5, 4)	0.648
Conjunctival chemosis	4	4 (2, 4)	4 (2.5, 4)	0.648
Corneal infiltration	4	3 (2, 4)	4 (1.75, 4)	0.527
Corneal oedema	4	4 (3, 4)	4 (2.5, 4)	0.788
**Total**	4	15 (10, 16)	16 (9.25, 16)	0.527

### Anterior segment optical coherence tomography

**Fig 4:**
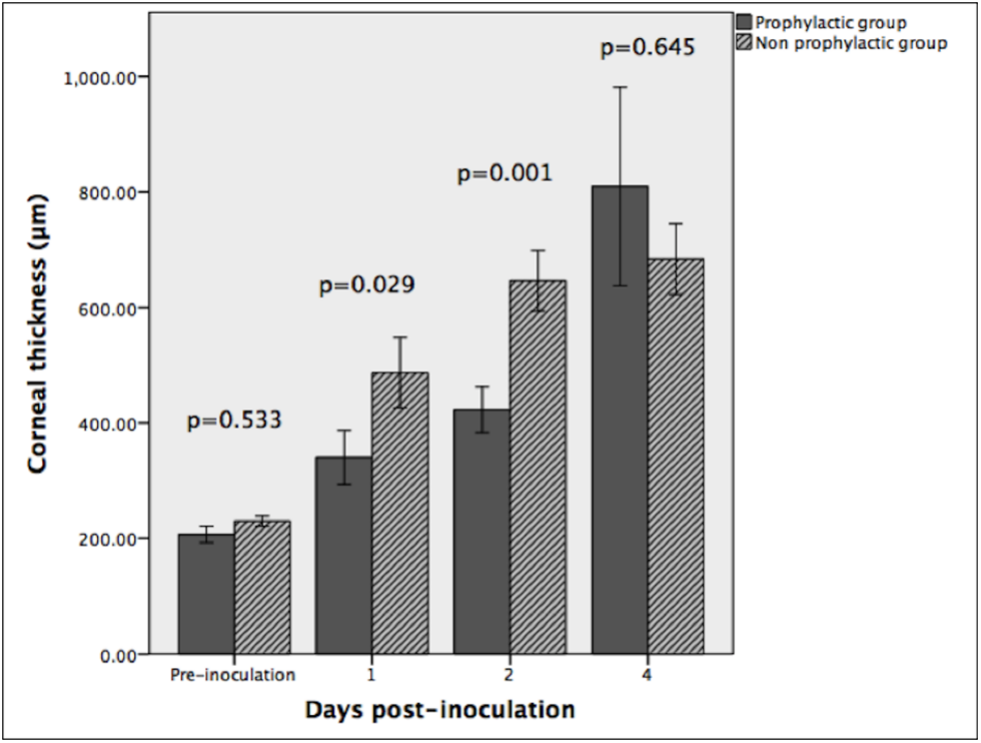
Corneal thickness. Comparison of corneal thickness between the prophylactic and non-prophylactic group. (error bars represent ± 1 standard error of mean)

Pre-inoculation (mean±SEM) CT was not significantly different between non-prophylactic and prophylactic groups (229.9±9.1 vs. 219.3±12.9 μm, p = 0.533). On day 1 post-inoculation, CT in the non-prophylactic group was significantly greater than in the prophylactic group (486.9±61.2 vs. 327.4±37.1 μm, p = 0.029). On day 2 post-inoculation, CT was also greater in the non-prophylactic group (646.2±52.6 vs. 404.7±36.2 μm, p = 0.001). On day 4, however, there was no significant difference between the non-prophylactic and prophylactic groups (683.8±61.2 vs. 765.7±146.9 μm, p = 0.645). The CT comparison between the two groups is illustrated in Fig 4.

In the non-prophylactic group, CT increased from 229.9±9.1 μm pre-inoculation to 486.9±61.2, 646.2±52.6 and 683.8±61.2 μm on days 1, 2 and 4 respectively (p<0.001). Bonferroni post-hoc tests showed that the difference was significant between pre-inoculation and day 1 CT (p = 0.003), but not between day 1 and day 2 CT (p = 0.128) or between day 2 and 4 (p = 1).

In the prophylactic group, CT increased from 219.3±12.9 μm pre-inoculation to 327.4±37.1, 400.4.7±36.2 and 765.7±146.9 μm on days 1, 2 and 4 respectively (p<0.001). Bonferroni post-hoc tests showed that, compared to before inoculation, the average CT was not significantly different on day 1 (p = 0.590) but became significantly different on day 2 (p = 0.050). Average CT was not significantly different between days 1 and 2 (p = 1.0) but was between days 2 and 4 (p<0.001).

### Bacterial quantification

**Fig 5:**
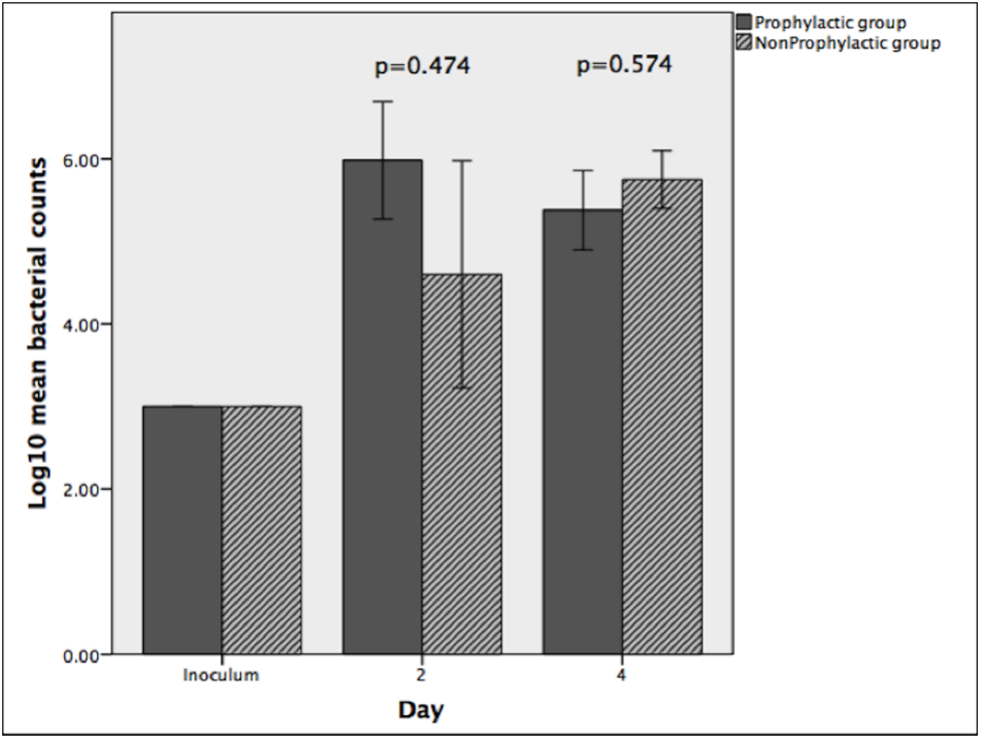
Bacterial quantification. Comparison of bacterial counts between the prophylactic and non-prophylactic group. (error bars represent ± 1 standard error of mean)

Log10 mean (±SEM) bacterial counts were not significantly different between non-prophylactic and prophylactic cases on day 2 (4.6±1.0 vs. 5.6±0.4 CFU/cornea, p = 0.474) and day 4 (5.7±0.2 vs. 5.4±0.5 CFU/cornea, p = 0.574). This is illustrated in Fig 5.

### Histology and immunohistochemistry

**Fig 6:**
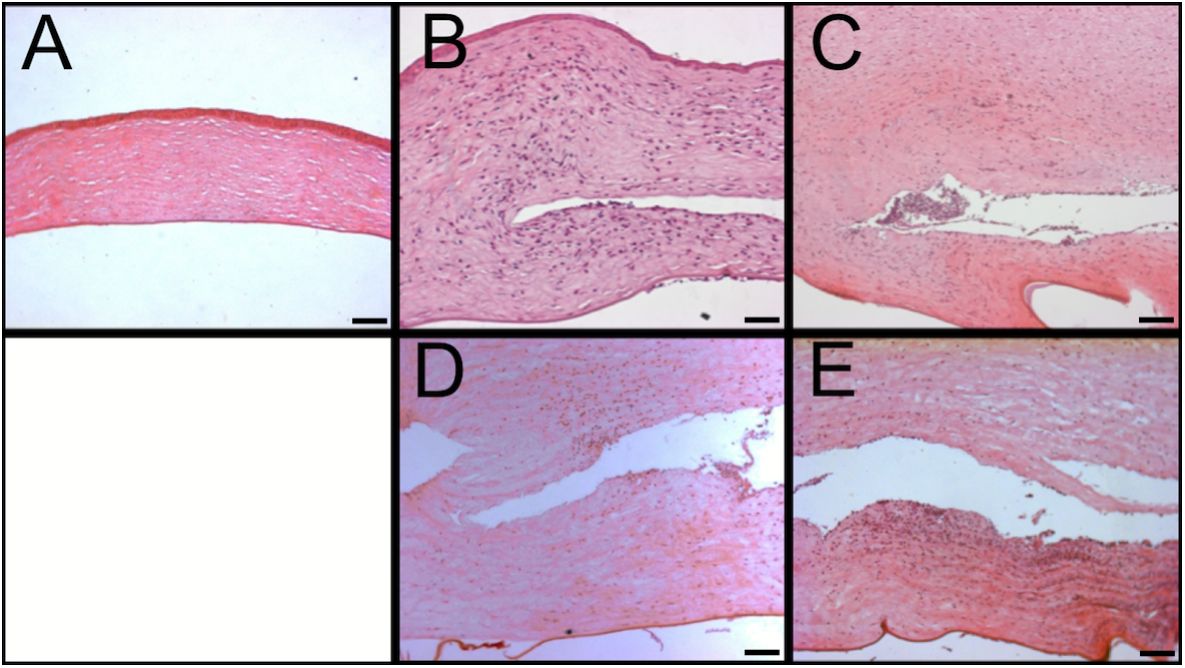
Histology. Haematoxylin and eosin stained corneal sections, comparing vancomycin prophylaxis to non-prophylaxis cases. A. Healthy cornea. B (day 2, prophylaxis). A rich infiltration of neutrophils is present throughout the stroma. The epithelial and endothelial surfaces are irregular due to stromal oedema. C (day 2, non-prophylaxis). The stroma appears more oedematous than in image B. A rich neutrophilic infiltration is also present, mostly in the pocket, but the neutrophils appear to be less densely arranged in the stroma than in image B, most likely reflecting the presence of more oedema. D (day 4, prophylaxis). The stroma is more oedematous than in image B and the Descemets membrane is detached, reflecting greater levels of infection. The neutrophilic infiltration is not as dense as in image B, most likely due to more stromal oedema. E (day 4, non-prophylaxis). The stroma appears similarly oedematous to that in image D and slightly richer in neutrophilic infiltration. (Scale bar 100μm)

The corneal stroma of rabbits that were euthanized on day 2 was thickened in both groups, with greater thickness present in the non-prophylactic group (Fig 6). The epithelial and endothelial surfaces appeared irregular and the stroma less densely populated by keratocytes compared to the healthy cornea. Numerous polymorphonuclear neutrophil (PMN) cells were present throughout the corneal stroma in both infection groups. The corneas of rabbits that were sacrificed on day 4 showed similar microscopic features but the stromal thickening and oedema appeared to be approximately equal in prophylactic and non-prophylactic cases.

**Fig 7:**
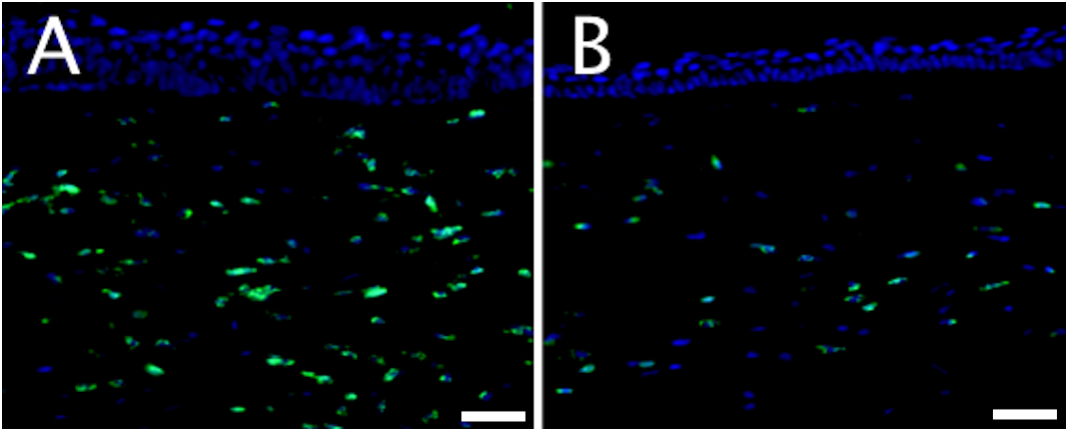
Immunohistochemistry. Comparison between prophylaxis and non-prophylaxis on day 2 following bacterial inoculation. More CD11b +ve neutrophils (fluorescing green) are present in the non-prophylactic case (A) than the prophylactic case (B). (Scale bar 50μm).

Immunostaining of day 2 corneal sections showed that the CD11b +ve/non-CD11b +ve cell ratio was greater in the non-prophylactic than in the prophylactic cases (1.45 vs. 0.71) (Fig 7). On day 4, the ratio was slightly larger in the non-prophylactic than prophylactic cases but the difference between the two was small (1.71 vs. 1.30).

### Pharmacokinetics and effectiveness of minimum inhibitory concentration of vancomycin

**Fig 8:**
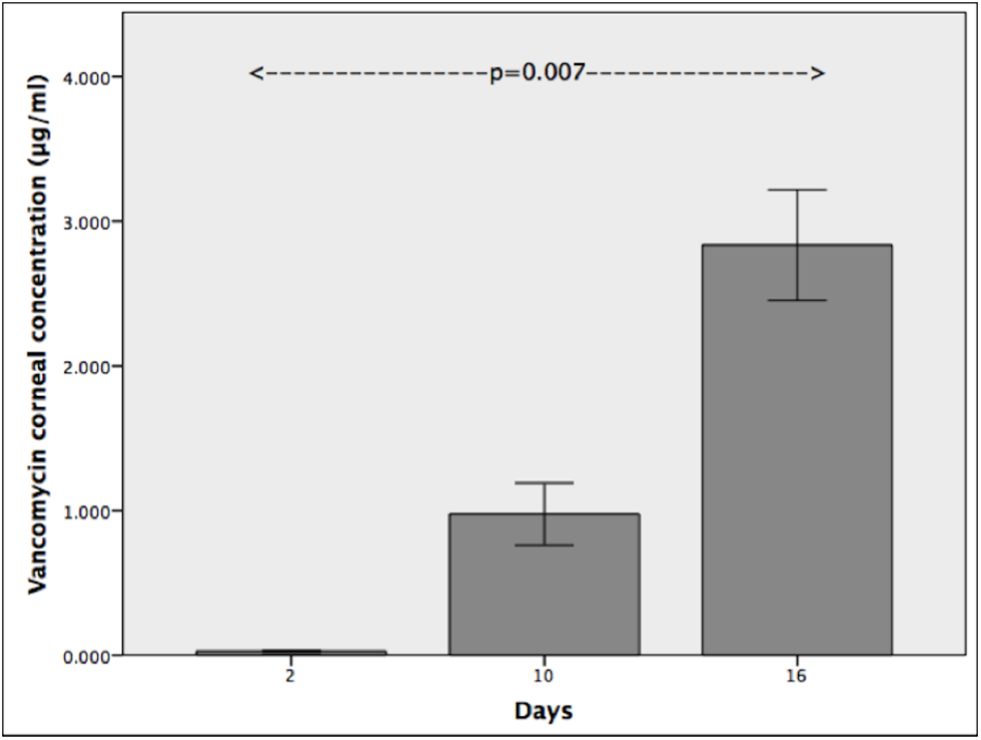
Vancomycin pharmacokinetics. Corneal vancomycin concentration increases with duration of vancomycin drop administration. (error bars represent ± 1 standard error of mean)

Corneal vancomycin concentration (mean±SEM) of rabbits that were sacrificed on days 2, 10 and 16 after initiation of vancomycin drops was 0.027±0.008, 0.975±0.215 and 2.835±0.383 μg/ml respectively (p = 0.007) (Fig 8). Vancomycin was not detected in the aqueous humour after 2 days of antibiotics; although it was detected in the day 10 and day 16 rabbits, it was below the level of quantification.

In the experiment that investigated the effect of removing the epithelium on corneal vancomycin concentration, the concentration after 4 days was 1.36±0.45 μg/ml without epithelial removal and 2.57±0.40 μg/ml with epithelial removal (p = 0.115). The aqueous humour vancomycin concentration was 0.04±0.005 μg/ml without epithelial removal and 0.43±0.15 μg/ml with epithelial removal (p = 0.063).

In the experiment that investigated the effectiveness of vancomycin prophylaxis once corneal vancomycin MIC for *Staphylococcus aureus* had been achieved, 2 of the 3 rabbits showed evidence of corneal infection with the development of an injected, sticky eye and a corneal infiltrate by day 2 post-inoculation. In the 3 rabbits, the CT (mean±SEM) increased from 228.8±5.7 μm before inoculation to 336.9±40.9 μm on day 1 and 431.7±47.2 μm on day 2 post-inoculation (p = 0.021).

## Discussion

Prophylactic vancomycin 1.4% drops, applied five times a day, reduced the severity of *S*. *aureus* bacterial keratitis for the first 2 days of infection compared to non-prophylaxis. However, they did not provide a sustainable benefit and they did not reduce the corneal bacterial counts. Corneal vancomycin levels remained below MIC for *Staphylococcus aureus* following 10 continuous days of vancomycin drop instillation, but did reach MIC by day 16. Achieving MIC did not prevent the development of corneal infection.

In this study we investigated the effectiveness of vancomycin drop prophylaxis, as patients with Boston type-1 Kpro typically receive once or twice daily prophylactic vancomycin drops with or without a fluoroquinolone.[17, 24] However, bacterial keratitis can still develop in patients receiving prophylaxis,[17] and the risk of fungal keratitis may even be increased.[24] The recommendation for the use of postoperative prophylactic vancomycin drops stems from the beneficial effect vancomycin drops have been found to have on the rate of endophthalmitis.[18,19] This potential benefit, however, may be confounded by other modifications to the treatment of Boston type-1 patients, such as the use of a bandage contact lens to keep the corneal surface hydrated,[18,19,25] and the redesign of the Kpo back-plate to include holes with the aim of improving nutrition of the corneal graft carrier.[26] Our study, although an animal model of Kpro infection involving a higher frequency of vancomycin drop use than typical regimens for Boston type-1 patients, suggests that vancomycin drops may only provide a limited prophylactic benefit.

The use of prophylactic vancomycin drops reduced the severity of the early corneal inflammatory response, but the efficacy was lost by day 4 following inoculation. Anterior segment optical coherence tomography provides morphology-based quantification of corneal inflammation. It can quantify the corneal inflammatory response in microbial keratitis with measurements of CT and monitor the progress of bacterial keratitis with serial measurements.[27,28] The AS-OCT CT was less in the prophylactic than the non-prophylactic group on days 1 and 2, but was not different between the groups on day 4. The initial efficacy of the prophylactic regimen is also supported by the fact that CT in the prophylactic group on day 1 was not significantly increased compared to pre-inoculation, but day 1 CT was increased in the non-prophylactic group. The SLP scores also showed an initial prophylactic benefit, as the day 1 total score was 1 in the prophylactic group compared to 11 in the non-prophylactic, but this was lost by day 2.

Immunohistochemistry and H&E microscopy also detected smaller levels of PMN infiltration in the prophylactic group on day 2, but there did not appear to be a difference on day 4. We found that the counts of viable bacteria were not reduced by vancomycin prophylaxis on days 2 and 4, providing further evidence that prophylactic drops may not provide a sustainable benefit. It is well documented that bacteria stimulate an innate immune response, rich in PMNs, via the interaction of their pathogen-associated molecular patterns with Toll-like receptors on corneal epithelial cells and stromal fibroblasts.[29–31]

Vancomycin drop instillation did not prevent the development of infection despite MIC levels for *S*. *aureus* being achieved by day 16 of drop use. In-vivo inhibition of bacteria may require a much higher concentration than the nominal MIC that is based on in-vitro tests, as the protease rich microenvironment of the cornea may degrade the vancomycin. Although there are no data for the cornea, it is known that vancomycin, a glycopeptide antibiotic, shows moderate binding to proteins. It may therefore bind to lectin-like proteins of the stromal extracellular matrix, reducing its bioavailability further.[32] These factors, combined with the slow bactericidal activity of vancomycin,[33] may account for its poor prophylactic effectiveness in our study. In view of this discrepancy between laboratory efficacy and clinical effectiveness for vancomycin, other broad-spectrum antibiotics may need to be investigated as suitable alternatives for prophylaxis.

Study of the corneal vancomycin pharmacokinetics showed that a period of 16 days with a strict drop instillation regimen of five times a day and 100% compliance was required to achieve MIC, unless the epithelium was debrided in which case MIC was achieved within 4 days. This raises the question whether the typical prophylactic use of antibiotic drops once or twice daily [17,24] would actually achieve MIC for a range of pathogens. Even if more frequent instillation was recommended, it is unlikely patients would remain compliant with a more intensive use over the long-term. There is good evidence from glaucoma studies that non-compliance with drops can be as high as 80%.[34] At only 3 months after starting treatment, just over half (55.6%) of patients took greater than 75% of the expected doses, even though once daily dosing was recommended.[35] Future developments, such as sustained drug release systems with liposomes and anti-infective or anti-adhesive biomaterials, may address these limitations.[36,37]

In conclusion, our animal model study of keratoprosthesis and corneal infection has shown that vancomycin drop instillation at a frequency that is higher than prophylactic clinical practice provided a short term benefit but did not prevent the development of *S*. *aureus* keratitis. In addition, even after the vancomycin MIC in the cornea was achieved, this was not sufficient to prevent corneal infection. Investigation of alternative antibiotic agents and of the discrepancy been laboratory efficacy and clinical effectiveness is required to improve our infection prophylaxis strategies in keratoprosthesis surgery. Despite the use of post-operative antibiotics, patients should continue to be counselled regarding the long-term risk of infection following keratoprosthesis surgery.
