# Correction: Prophylactic Vancomycin Drops Reduce the Severity of Early Bacterial Keratitis in Keratoprosthesis

**DOI:** 10.1371/journal.pone.0145941

**Published:** 2015-12-22

**Authors:** The PLOS ONE Staff

The tenth author’s name is incorrectly spelled. The publisher apologizes for the error. The correct name is: JS Mehta.

## References

[1] Konstantopoulos A, Tan XW, Goh GTW, Saraswathi P, Chen L, Nyein CL, et al (2015) Prophylactic Vancomycin Drops Reduce the Severity of Early Bacterial Keratitis in Keratoprosthesis. PLoS ONE 10(10): e0139653 doi:10.1371/journal.pone.0139653 26460791 10.1371/journal.pone.0139653 PMC4604170

